# Comparative transcript profiling of alloplasmic male-sterile lines revealed altered gene expression related to pollen development in rice (*Oryza sativa* L.)

**DOI:** 10.1186/s12870-016-0864-7

**Published:** 2016-08-05

**Authors:** Jihong Hu, Guanglong Chen, Hongyuan Zhang, Qian Qian, Yi Ding

**Affiliations:** State Key Laboratory of Hybrid Rice, College of Life Sciences, Wuhan University, Wuhan, 430072 China

**Keywords:** Cytoplasmic male sterility, *Oryza sativa* L., Microarray, Sporophytic, Gametophytic, Pollen development

## Abstract

**Background:**

Cytoplasmic male sterility (CMS) is an ideal model for investigating the mitochondrial-nuclear interaction and down-regulated genes in CMS lines which might be the candidate genes for pollen development in rice. In this study, a set of rice alloplasmic sporophytic CMS lines was obtained by successive backcrossing of Meixiang B, with three different cytoplasmic types: D62A (D type), ZS97A (WA type) and XQZ-A (DA type).

**Results:**

Using microarray, the anther transcript profiles of the three *indica* rice CMS lines revealed 622 differentially expressed genes (DEGs) in each of the three CMS lines compared with the maintainer line Meixiang B. GO and MapMan analysis indicated that these DEGs were mainly involved in lipid metabolism and cell wall organization. Compared with the gene expression of sporophytic and gametophytic CMS lines, 303 DEGs were identified and 56 of them were down-regulated in all the CMS lines of rice. These down-regulated DEGs in the CMS lines were found to be involved in tapetum or cell wall formation and their suppressed expression might be related to male sterility. Weighted gene co-expression network analysis (WGCNA) revealed that two modules were significantly associated with male sterility and many hub genes that were differentially expressed in the CMS lines.

**Conclusion:**

A large set of putative genes involved in anther development was identified in the present study. The results will give some information for the nuclear gene regulation by different cytoplasmic genotypes and provide a rich resource for further functional research on the pollen development in rice.

**Electronic supplementary material:**

The online version of this article (doi:10.1186/s12870-016-0864-7) contains supplementary material, which is available to authorized users.

## Background

Cytoplasmic male sterility (CMS) is a maternally inherited trait that cytoplasmically determines the inability to produce functional pollen in flowering plant [1]. CMS cytoplasm has often been discovered in wild germplasm or is obtained by inter- or intra-subspecies backcrossing [2]. In China, the success in breeding hybrid rice cultivars has been largely due to the utilization of CMS resources without laborious emasculation [3]. In the past few decades, many types of CMS lines have been developed and used in hybrid breeding in China, such as wild abortive (WA) type, dwarf-wild-abortive (DA) type, Honglian (HL) type, Dissi (D) type, Indonesia paddy (ID) type and Maxie type and so on [4, 5]. Many of these CMS lines are sporophytic and their pollen grains are irregularly shaped and unstainable with 1 % I_2_–KI solution [4]. One of the WA-CMS lines is the ZS97-A which has been widely used in the hybrid rice breeding in the past years. Compared with ZS97-A, the D62-A (D type) CMS line has more second branches. In addition, Xieqingzao A (XQZ-A), a DA-CMS line, originated from dwarf wild rice in Jiangxi, China and its anther is small without cracking [6].

In addition to their commercial use, CMS studies contribute to a better understanding of the incompatibilities of the nuclear–mitochondrial intracellular genomic barrier. It is well documented that a specific cytoplasm containing an unusual chimeric open reading frame (ORF) in the CMS mitochondrial genome is responsible for male sterility [7]. Recently, the mechanism of male sterility in some rice CMS systems was well studied, and the chimeric ORFs were identified. Rice BT-CMS has been known to contain a CMS-associated cytotoxic peptide ORF79, which co-transcribes with an additional copy of *atp6* (B-*atp6*) [8]. In WA-CMS, a new mitochondrial gene, *WA352*, which accumulated preferentially in the anther tapetum, was found to be associated with male sterility. The WA352 inhibited nuclear-encoded mitochondrial protein COX11 and triggered premature tapetal programmed cell death and consequent pollen abortion [9].

In recent years, a new type of CMS line, ZidaoA (ZD-CMS) was developed from the cytoplasm of Yunnan purple rice (patent No. ZL 99 1 20003.9 in China). The pollen abortion of ZidaoA occurred earlier than in other CMS lines such as ZS97A (WA-CMS), YtA (HL-CMS) and MaxieA (MX-CMS), which was revealed by multispectral imaging analysis [10, 11]. Cytological studies of the CMS line MA (ZD-CMS) indicated that the pollens were aborted at the uninucleate microspore stage [11–13]. The CMS line Meixiang A (MA) was derived from the ZD-CMS system by successive backcrossing, and its corresponding maintainer line was Meixiang B (MB) [12, 14]. Previous studies reported that different RNA editing patterns of *atp*9 between MA and MB led to an arginine codon to a termination codon in MB, and the RNA editing events of *cox*2, *atp*6 and *atp*9 were affected by nucleo-cytoplasmic interactions [14, 15]. Quantitative proteomics of MA and MB at uninucleate microspore stage by iTRAQ-based approach showed that the proteins for the stress response and carbohydrate metabolism were down-regulated in MA [12].

Although CMS is mainly caused by a mitochondrial chimeric ORF, the mechanism of cytoplasmic-specific dysfunctions in CMS remains unresolved. CMS is an ideal model for investigating mitochondrial-nuclear interactions and discovering the genes that are essential for pollen development [4]. Therefore, analysis of downstream reactions under CMS conditions will give some insights into mitochondrial-nuclear incompatibility. Additionally, the significant differentially expressed genes between the CMS lines and the maintainer line might be candidate genes for pollen development in rice. In this study, a set of alloplasmic CMS lines were obtained by successive backcrossing of MB, with three different cytoplasmic types: D62A (D type), ZS97A (WA type) and XieQingZao-A (DA type). By comparing the anther transcript profiles of the three *indica* rice CMS lines, the present study will provide information on nuclear gene regulation by different cytoplasmic genotypes.

## Results

### Dynamic reorganization of the three CMS mitochondrial genomes

**Fig. 1:**
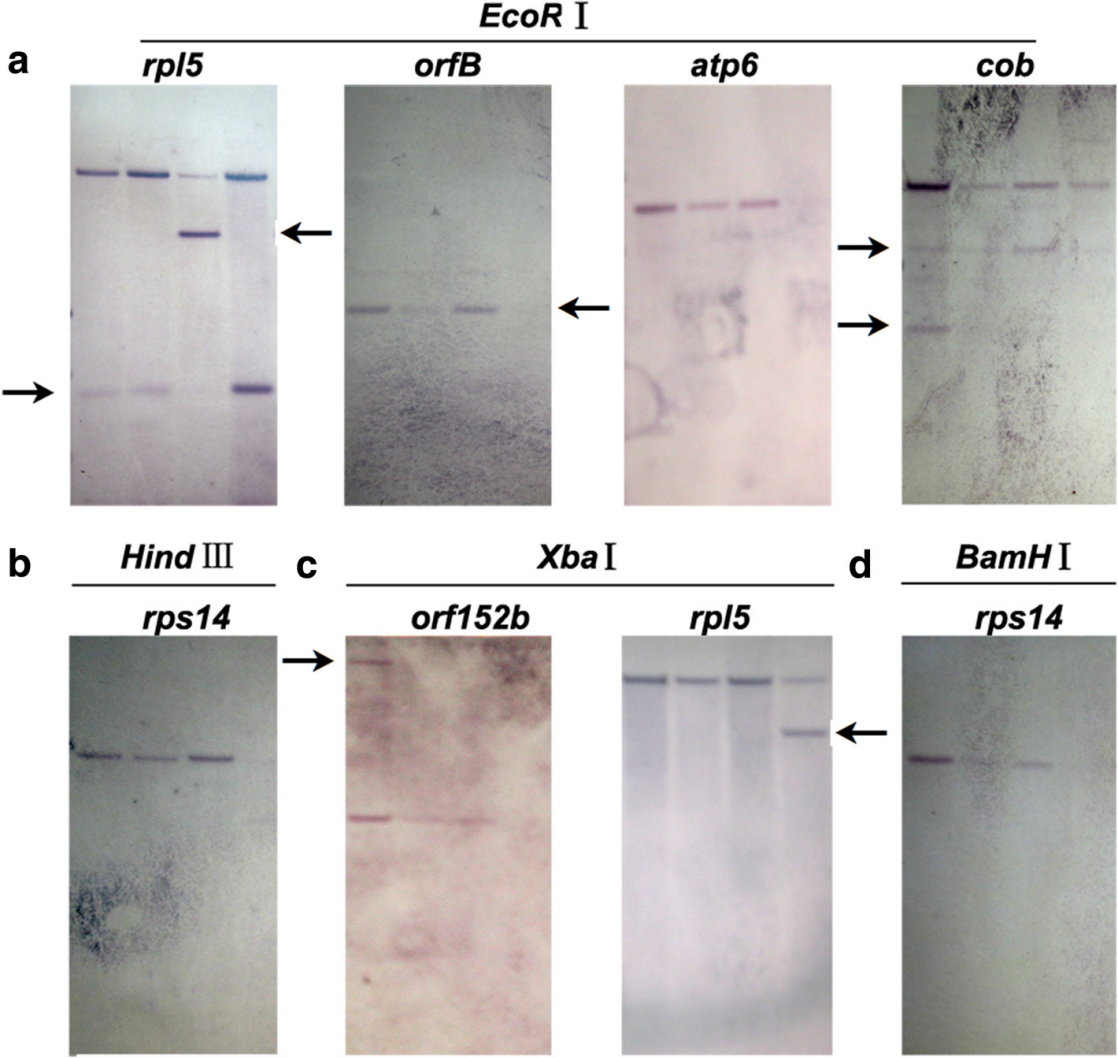
Comparison of the mitochondrial genomic structure of three CMS lines and the maitainer line by Southern blotting analysis. For each restricti on enzyme, from left to right are XQZ-A/MB, ZS97-A/MB, D62-A/MB, Meixiang B (MB). Polymorphisms of the detected probes were shown here. **a** *EcoR* I, **b** *Hind* III, **c** *Xba* I, and **d** *BamH* I

To show the mitochondrial genetic differences among the CMS cytoplasms, Southern blotting analysis were performed using probes designed from 6 rice mitochondrial genes (*atp6*, *cob*, *orfB*, *orf152b*, *rpl5* and *rps14*). In these genes, we detected RFLPs among the three CMS lines XQZ-A/MB, D62-A/MB, ZS97-A/MB and the maintainer line MB (Fig. 1). And the polymorphisms were also found among the CMS lines in four genes *rpl5*, *orfB*, *cob* and *orf152b* (Fig. 1a and c). These results showed that mitochondrial genomic organization was different among the three CMS lines and MB. Thus, we could further determine whether mitochondrial genome polymorphisms affected nuclear gene expression.

### Cytological characteristics of the CMS lines

**Fig. 2:**
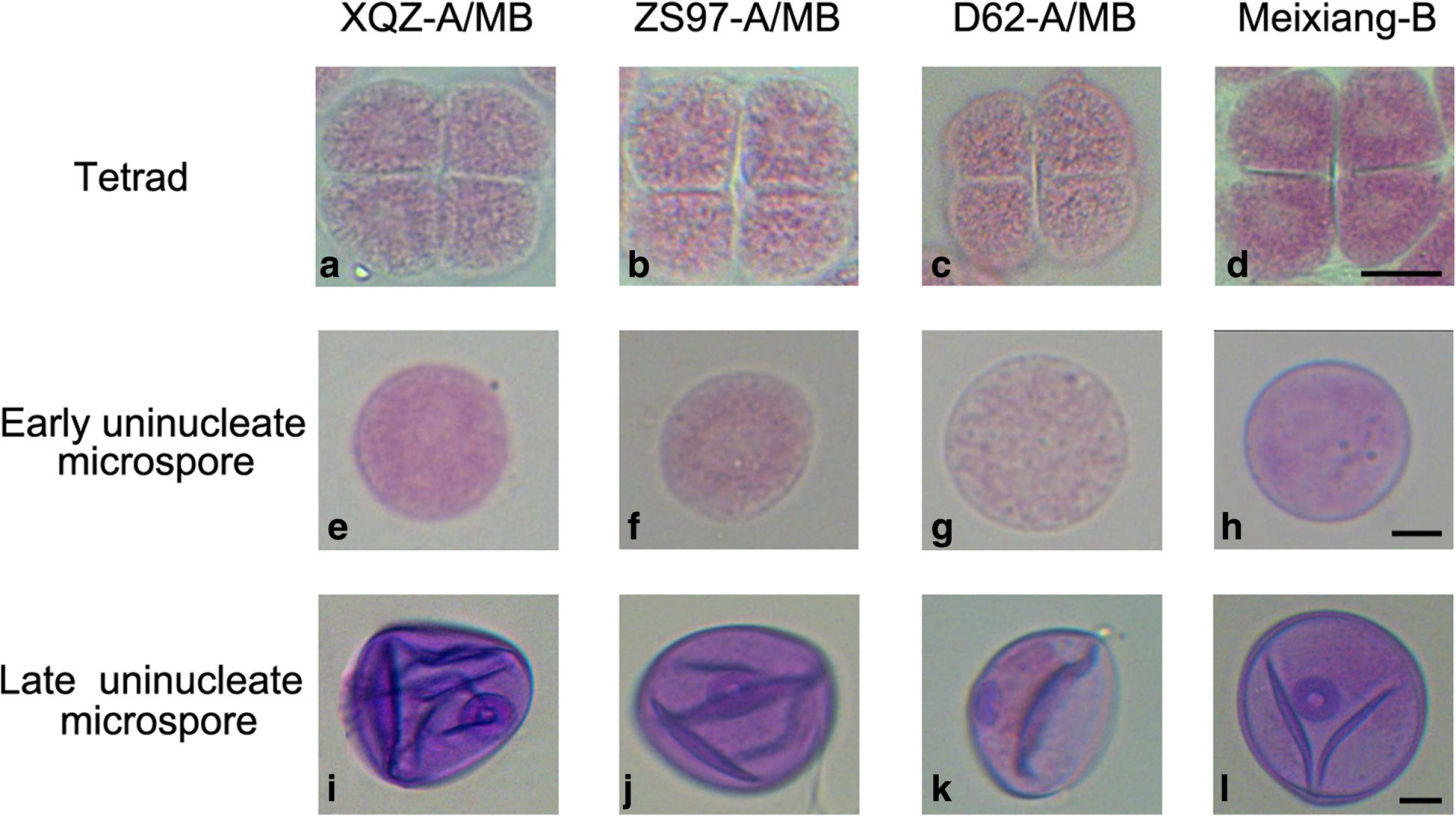
Cytological observation of microspores. The microspore phenotypes of the three CMS lines and the maintainer line Meixiang B at tetrad, early and later uninucleate microspore stages. **a**-**e**-**i**, **b**-**f**-**j**, **c**-**g**-**k** and **d**-**h**-**l** represent the microspore phenotypes of XQZ-A/MB, ZS97-A/MB, D62-A/MB and Meixiang-B at tetrad, early uninucleate microspore, and late uninucleate microspore, respectively

According to the phenotypes of the abortive pollens, the rice CMS system is generally categorized into sporophytic and gametophytic types. Sporophytic male sterility occurs relatively earlier during microspore development, mainly at the uninuleate stage, while gametophytic CMS lines abort at the dinucleate or mature pollen stages [12, 16]. In this study, three sporophytic CMS lines, DA-CMS, WA-CMS and D-CMS, were chosen to compare the phenotypes of the male reproductive tissues to conduct transcriptomic studies. Morphologically, anthers of the three CMS lines were light yellow (Additional file 1: Figure S1 A-C), and their microspores exhibited shrunken pollen phenotypes which were unstainable by I_2_-KI, indicating a lack of starch accumulation (Additional file 1: Figure S1 E-G). However, the anthers of the maintainer line MB were darkly yellow (Additional file 1: Figure S1 D), and an apparent starch accumulation was observed by I_2_-KI (Additional file 1: Figure S1 H). To monitor microspore development, the pollen phenotypes of the CMS lines from tetrad to uninucleate microspores were observed with improved carmine staining. At the tetrad stage, all the CMS lines could yield normal tetragonal tetrads and four haploid daughter cells (Fig. 2a-d). Each of the early uninucleate microspores was also normal (Fig. 2e-h), however, at the later uninucleate microspore stage, all three CMS lines exhibited complete pollen dysfunction (Fig. 2i-k) and only the maintainer line MB had mature pollen grains (Fig. 2l).

### Differential gene expression in the three CMS lines

**Fig. 3:**
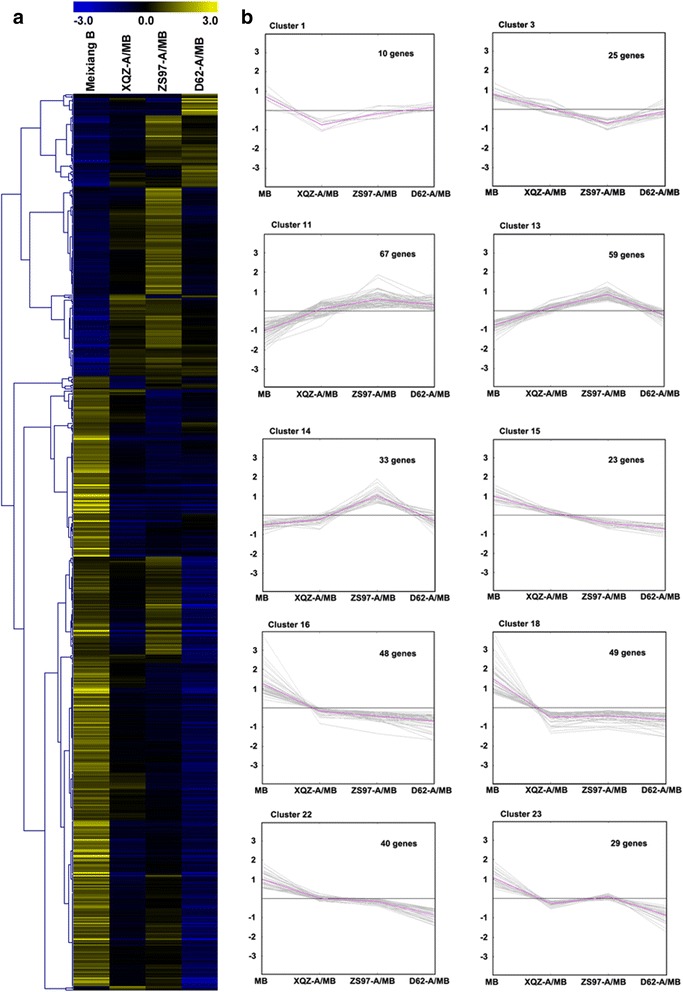
Clustering analysis of 622 DEGs in the three CMS lines and the maintainer line Meixiang B. **a** Hierarchical cluster of the 622 DEGs in each of the CMS lines and Meixiang B. **b** Accumulation patterns of some clusters of the 622 DEGs in different CMS lines and Meixiang B

To understand the gene expression profiles in the three CMS lines, microarray experiments were conducted with a GenAtlas Rice (Cn) Gene 1.1 ST Array Strip (ssp. *Indica*). Of the total 40,565 probes, 1187, 1697 and 1481 differential probes with at least two-fold change were identified in the three CMS lines XQZ- A/MB, ZS97-A/MB and D62-A/MB, compared to MB, respectively. After being annotated by the RGAP database (http://rice.plantbiology.msu.edu/), a total of 622 differentially expressed genes (DEGs) were found in any one of the three CMS lines and 114 genes of them were shared by the three CMS lines (Fig. 3, Additional files 2, 3, 4 and 5: Figure S2, Figure S3, Table S1 and Table S2). Hierarchical clustering of the nuclear gene expression profiles of the MB and the CMS lines showed different expression patterns among them (Additional files 3 and 4: Figure S3 and Table S1). Most of the shared genes were down-regulated in the CMS lines, including chalcone synthase (LOC_Os11g32650), WAX2 (LOC_Os10g33250), cytochrome P450 (LOC_Os08g03682, LOC_Os04g48210, LOC_Os04g33370, LOC_Os03g07250), and Invertase/pectin methylesterase inhibitor (PMEI, LOC_Os06g49760). These genes mainly participated in pollen exine formation. Previous studies reported that chalcone synthase (CHS) synthesizes naringenin chalcone and the inhibition of CHS leads to male sterility in maize and petunia [17, 18]. In Arabidopsis, the WAX2 gene was documented to involve in cuticle membrane and wax production [19]. PMEIs have been thought to be regulators of cell wall stability at the tip of the pollen tube and were demonstrated to participate in pollen tube growth in *Brassica oleracea* [20]. These down-regulated genes in the three CMS lines, will affect pollen exine formation during anther development.

**Fig. 4:**
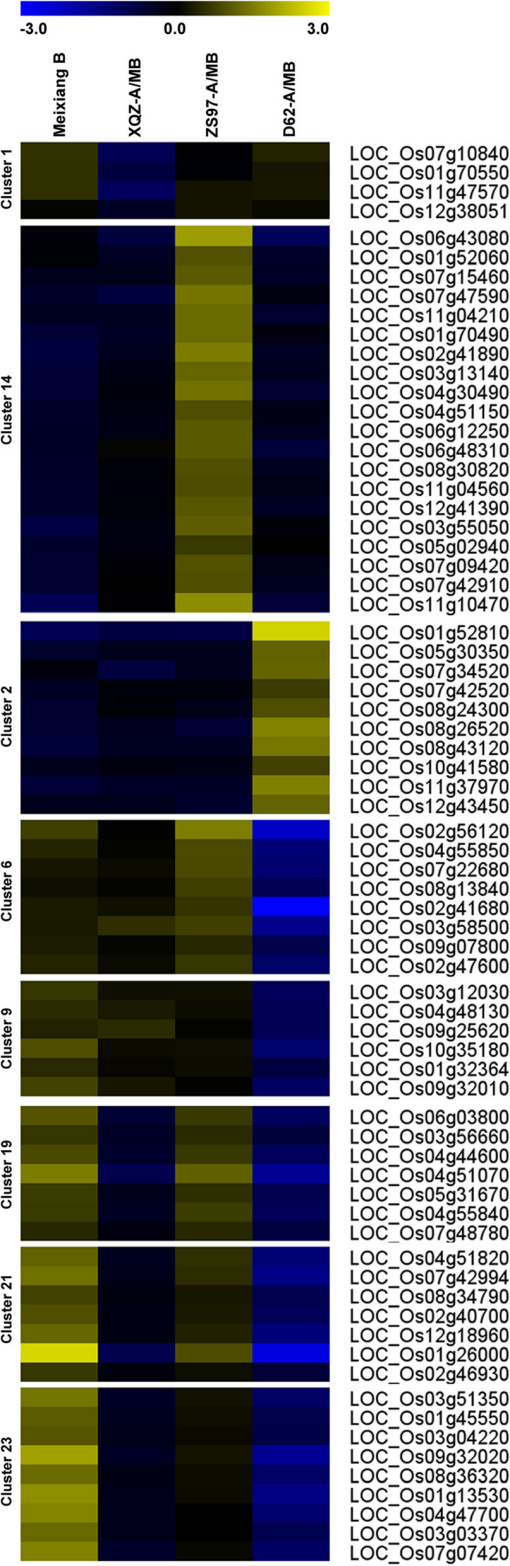
Visualization of relative expression levels of the representative gene clusters. Expression levels are presented as normalized log_2_ values

Using k-means clustering, these 622 DEGs were categorized into 25 clusters, and many of the DEGs were highly expressed in MB (Figs. 3 and 4 and Additional file 4: Table S1). For example, clusters 3, 4, 12, 15, 16, 17, 18 and 22 showed high expression levels in MB. These genes included protein kinase, hormone-related genes, ATPase-related genes, genes related to pollen development and cell wall organization (Additional file 4: Table S1). Cluster 12 and cluster 16 included genes such as cytochrome P450 (LOC_Os04g48210, LOC_Os03g04650, LOC_Os03g07250 and LOC_Os08g03682), male sterility protein (LOC_Os03g07140), ribosome inactivating protein (LOC_Os07g37090), WAX2 (LOC_Os10g33250), plasma membrane ATPase (LOC_Os12g44150), mitochondrial TIM17 (LOC_Os05g02060), and 2Fe-2S iron-sulfur cluster (LOC_Os08g01380) which are related to pollen development and cell wall organization or energy synthesis (Additional file 4: Table S1). All the genes were suppressed in the three CMS lines, which indicated that they may be associated with male sterility. In contrast, clusters 10 and 11 were the genes suppressed in the MB, while they were expressed at a higher level in each CMS line (Additional file 4: Table S1). Glutathione S-transferase (LOC_Os10g38340 and LOC_Os08g44400), which was reported to produce a reactive oxygen species (ROS) response, was highly expressed in the three CMS lines in comparison with the maintainer line MB. In addition, two WRKY family genes, WRKY76 (LOC_Os09g25060) and WRKY104 (LOC_Os11g02520) were also up-regulated in the CMS lines.

These genes, however, could be distinguished by identifying different nuclear gene clusters in this study (Fig. 4). Cluster 1 and cluster 4 were the genes that were significantly down-regulated in the XQZ-A/MB specifically (Additional file 4: Table S1). Genes in cluster 14 were aberrantly highly expressed in ZS97-A/MB. These genes included ATPase (LOC_Os05g02940 and LOC_Os07g09420) and cytochrome c oxidase (LOC_Os07g42910). Furthermore, genes down-regulated specifically in ZS97-A/MB were found in cluster 3 (Additional file 4: Table S1). Genes in cluster 2 were up-regulated in D62-A/MB and clusters 6, 9 and 20 were the genes down-regulated only in D62-A/MB (Additional file 4: Table S1). Therefore, we identified some nuclear markers that could distinguish the cytoplasmic-nuclear gene regulation during male sterility in each CMS line. Interestingly, the suppressed genes in XQZ-A/MB and D62-A/MB showed similar patterns. For instance, genes in clusters 8, 19, 21, 23, and 25 were both down-regulated in XQZ-A/MB and D62-A/MB compared with the maintainer line MB or the CMS line ZS97-A/MB (Additional file 4: Table S1). The results indicated that DA-CMS and D-CMS may have a similar mechanism for pollen abortion, which was different from WA-CMS.

### Gene ontology and MapMan pathway analysis of DEGs

**Fig. 5:**
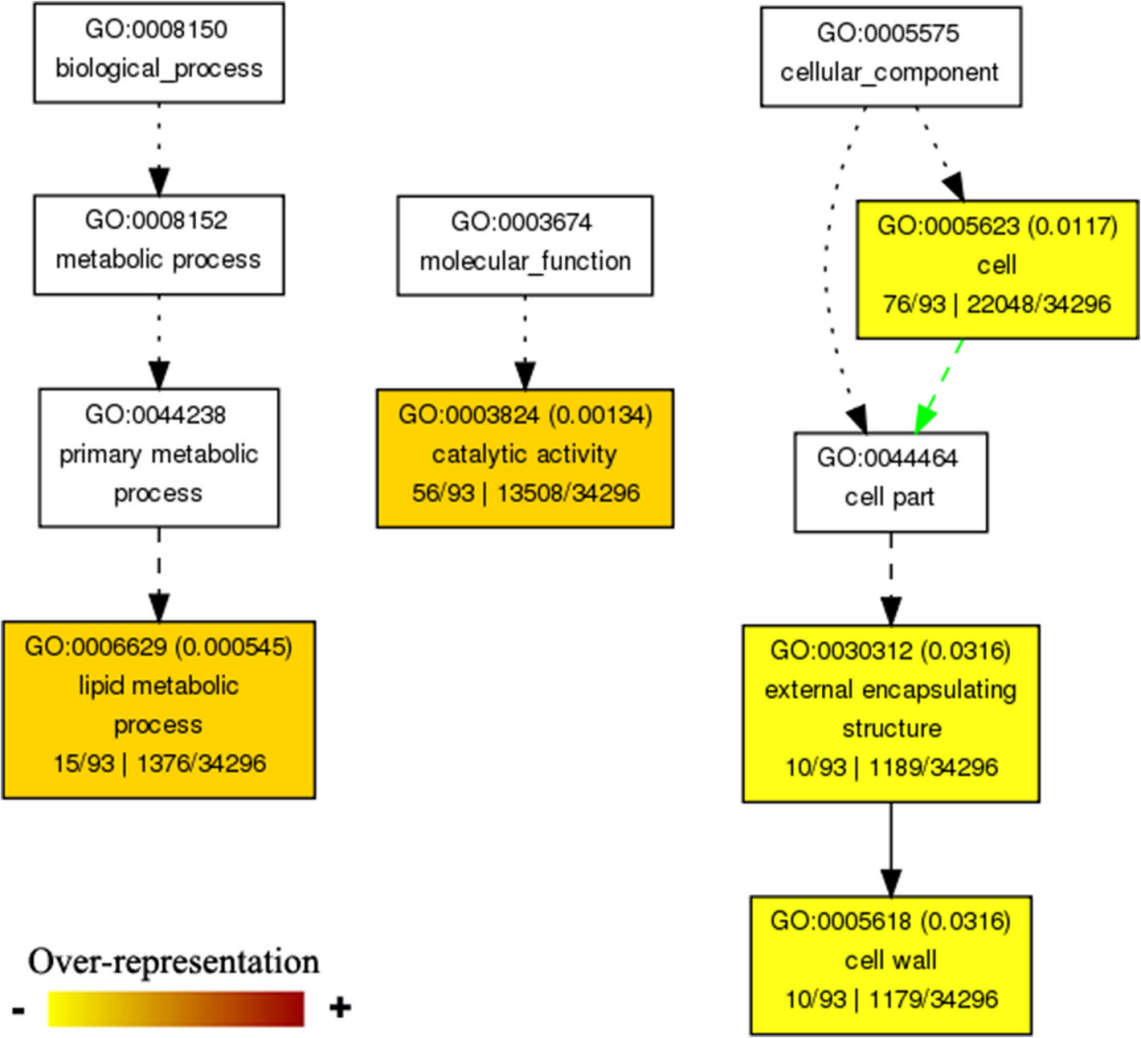
Venn diagram and AgriGO analysis of DEGs in the three CMS lines. AgriGO representation of the overrepresented GO terms in the 114 DEGs of the three CMS lines was generated using singular analysis enrichment (Fisher’s test, *P* < 0.05, FDR < 0.05). The number in parenthesis represents the FDR value

**Fig. 6:**
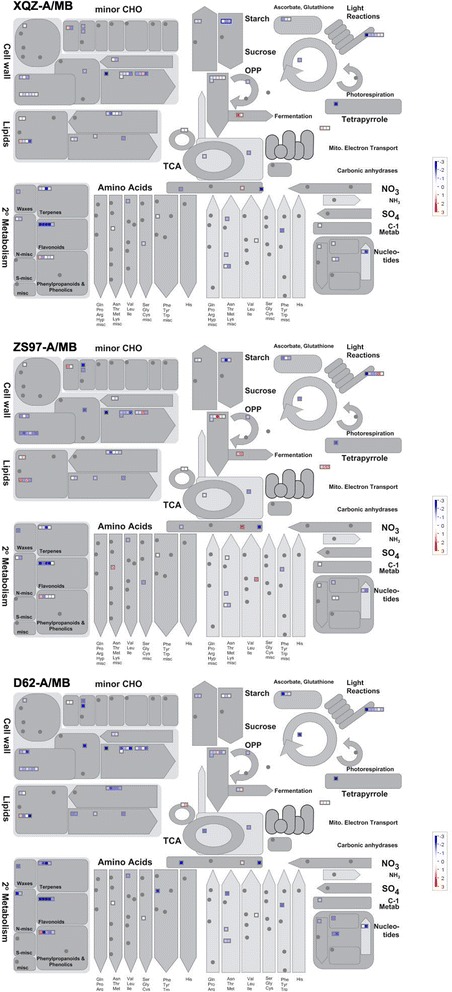
Overview of the metabolism of the three CMS lines by MapMan. The colour key represents normalized log_2_ values. Red represents up-regulation and blue represents down- regulation between the CMS line and maintainer line

To further examine the gene functional differences between the CMS lines and the maintainer line (MB), all 622 DEGs were annotated and subjected to GO and MapMan pathway analysis. Enrichment of the 622 DEGs showed that 36 significant GO terms were found (*p* < 0.01) (Additional file 6: Figure S4). These DEGs were mainly involved in metabolic process (including lipid and carbohydrate metabolic process), cell wall organization or biogenesis, transport (metal ion transport) and response to stimulus. Catalytic activity and binding (metal ion binding) was dominant in the category of molecular function (Additional file 3: Figure S3). Furthermore, GO analysis of the shared 114 DEGs in the three CMS lines showed that these genes were mainly involved in lipid metabolic processes and cell wall formation (Fig. 5). MapMan analysis also showed that many genes were associated with cell wall, lipid and metabolism processes (Fig. 6). The results indicated that pollen abortion for the three CMS lines might be related to lipid and carbohydrate metabolic processes and accumulated reactive oxygen species (ROS). In addition, the DEGs were mainly genes related to transcription factors, protein degradation and kinases, and some hormones, such as IAA, ABA, GA and ethylene. However, the expression levels of these genes were different in the three CMS lines.

### Validation of differentially expressed genes in the three CMS lines

**Fig. 7:**
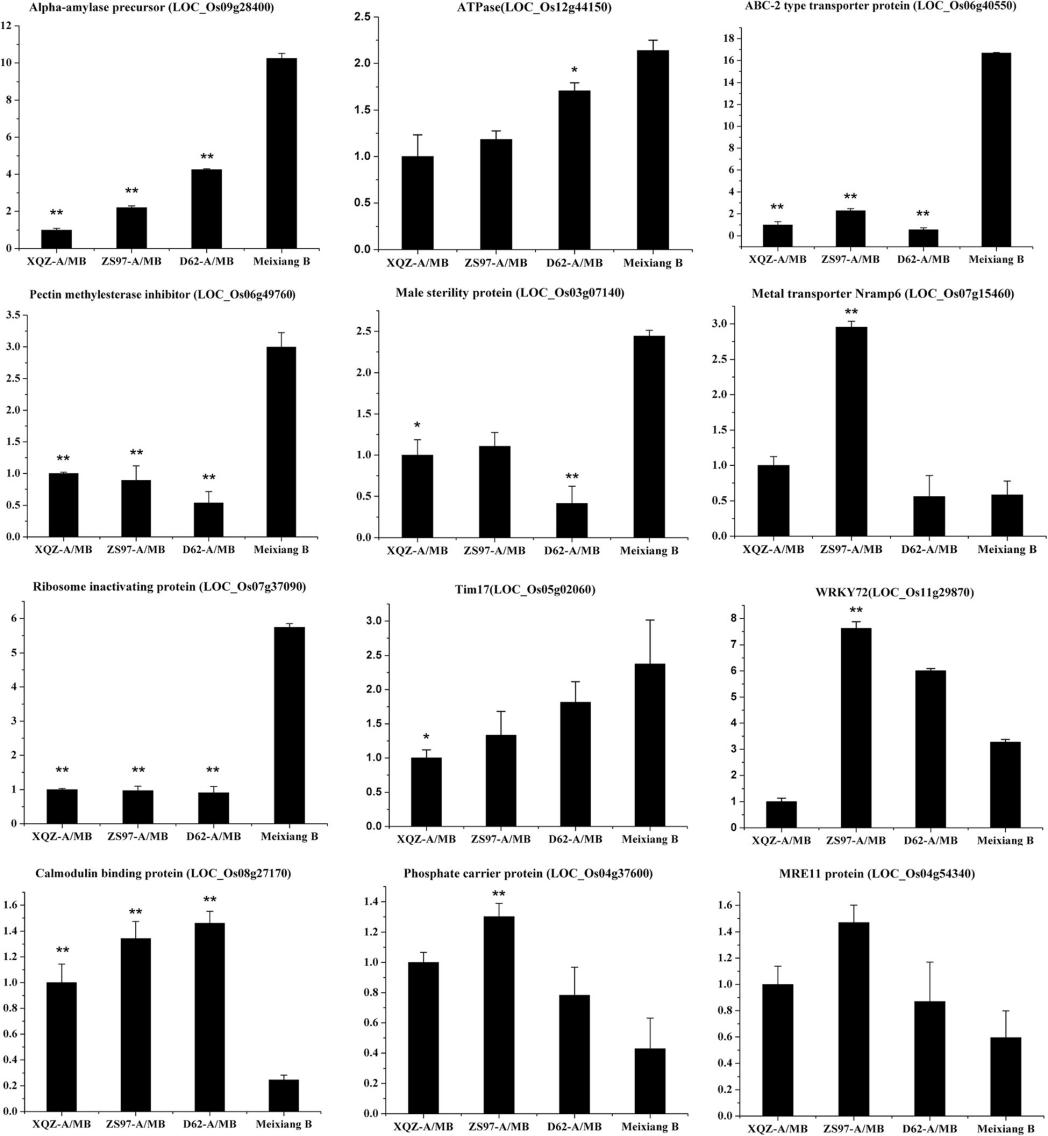
Identification of the expression level of 12 selected DEGs from the microarray by qPCR. Alpha-amylase precursor (LOC_Os0928400), ATPase (LOC_Os12g44150), ABC-2 type transporter protein (LOC_Os06g40550), pectin methylesterase inhibitor (LOC_Os06g49760), male sterility protein (LOC_Os03g07140), metal transporter Nramp6 (LOC_Os07g15460), ribosome inactivating protein (LOC_Os07g37090), Tim17 (LOC_Os05g02060), WRKY72 (LOC_Os11g29870), calmodulin binding protein (LOC_Os08g27170), phosphate carrier protein (LOC_Os04g37600), and MRE11 protein (LOC_Os04g54340). The significant differences of expression level between the CMS line and maintainer line were evaluated using Student's *t* test (* *p* < 0.05; ** *p* < 0.01)

To verify the microarray data and clarify their expression patterns, we conducted RT-qPCR on twelve selected genes that were differentially expressed between the three CMS lines and their maintainer line (Fig. 7). Pollen development related genes, such as alpha-amylase (LOC_Os09g28400), ABC-2 type transporter protein (LOC_Os06g40550), invertase/pectin methylesterase inhibitor (LOC_Os06g49760), male sterility protein (LOC_Os03g07140) and ribosome inactivating protein (LOC_Os07g37090) were suppressed in the three CMS lines (Fig. 7). Mitochondrial TIM17, which is a mitochondrial import inner membrane translocase subunit, was also expressed at a low level in the CMS lines [21]. However, three selected genes, including calmodulin binding protein (LOC_Os08g27170), phosphate carrier protein (LOC_Os04g37600) and MRE11 protein (LOC_Os04g54340) were verified to be up-regulated in the CMS lines (Fig. 7). Furthermore, the genes metal transporter Nramp6 (LOC_Os07g15460) and WRKY72 (LOC_Os11g29870) showed higher expression levels in ZS97-A/MB than the others (Fig. 7). These results indicated that the nuclear gene expression of WA-CMS was different from that of DA-CMS or D-CMS and had a specific abortion mechanism. These close correlations between the microarray data and the RT-qPCR validations highlighted the reliability of the microarray results used in the present study.

### Comparison of gene expression between sporophytic and gametophytic CMS lines

In rice, sporophytic male sterility always occurs relatively earlier than the gametophytic CMS line abortions, and they have different abortion mechanisms. In order to understand the difference between the two CMS types in rice, we compared the gene expression of the microarray results with those reported by Fujii et al. [2]. In the total of 622 DEGs, we found that 303 genes were also altered their expression in the CMS lines described by Fujii et al. [2]. Of these, 55 and 37 genes which showed the same patterns were up-regulated and down-regulated in the three CMS lines in the present study, respectively (Table 1 and Additional file 7: Table S3). The gametophytic CMS lines reported in the previous study included BT-CMS, CW-CMS, LD-CMS, and W11-CMS and their pollen phenotypes were normal until the tricellular pollen stage [2]. These results indicated that the sporophytic and gametophytic CMS lines might regulate some common genes during pollen abortion, though their abortion mechanisms were different. For instance, pyruvate kinase (LOC_Os01g47080), ABC-2 type transporter (LOC_Os06g40550), male sterility protein (LOC_Os03g07140), and ribosome inactivating protein (LOC_Os07g37090) were all strongly down-regulated in both sporophytic and gametophytic CMS lines (Additional file 7: Table S3). Moreover, the cytochrome c oxidase subunit (LOC_Os07g42910) and phosphate carrier protein (LOC_Os04g37600) showed high expression levels in all the CMS lines.

**Table 1:** Significantly differentially expressed genes between each of the three CMS lines and the maintainer line Meixiang B (MB)

RGAP_ID	RAPDB_ID	Annotation	XQZ-A/MB	ZS97-A/MB	D62-A/MB	Meixiang B
Up-regulated in MB						
LOC_Os01g07560.1	Os01g0170300	receptor-like protein kinase 2 precursor	−0.2069	−0.5904	−1.048	1.845
LOC_Os01g66330.1	Os01g0886600	ATP-dependent Clp protease ATP-binding subunit clpX	−0.5614	−0.3908	−0.8934	1.8455
LOC_Os02g01980.1	Os02g0110000	GDSL-like lipase/acylhydrolase, putative, expressed	−1.3225	−1.1067	−1.517	3.9465
LOC_Os03g03370.1	Os03g0125100	fatty acid hydroxylase, putative, expressed	−0.3472	0.032	−0.9512	1.2665
LOC_Os03g07250.1	Os03g0168600	cytochrome P450, putative, expressed	−0.5027	−0.5707	−1.6895	2.7625
LOC_Os03g14010.1	Os03g0243700	glycosyl hydrolase family 10 protein, putative, expressed	−0.1561	−0.4239	−0.8669	1.447
LOC_Os04g33370.1	Os04g0407900	cytochrome P450, putative, expressed	−0.2626	−0.4836	−0.4772	1.2235
LOC_Os04g48210.1	Os04g0570600	cytochrome P450, putative, expressed	−0.9813	−0.9355	−0.6901	2.607
LOC_Os05g34700.1	Os05g0419800	GDSL-like lipase/acylhydrolase, putative, expressed	−0.2070	−0.3188	−0.6501	1.1755
LOC_Os06g05550.1	Os06g0148200	GDSL-like lipase/acylhydrolase, putative, expressed	−0.3661	−0.0609	−0.6714	1.0983
LOC_Os06g48170.1	Os06g0696500	glycosyl hydrolases family 16, putative, expressed	−0.0667	−0.3345	−0.6643	1.0657
LOC_Os06g49760.1	Os06g0711800	invertase/pectin methylesterase inhibitor family protein	0.03012	−0.5528	−0.7377	1.2605
LOC_Os08g03682.1	Os08g0131100	cytochrome P450, putative, expressed	−0.2918	−0.4609	−1.0019	1.7545
LOC_Os08g45150.1	Os08g0565900	GDSL-like lipase/acylhydrolase, putative, expressed	−1.6005	0.9628	−1.95	2.5875
LOC_Os10g33250.1	Os10g0471100	WAX2, putative, expressed	−0.1448	−0.4345	−0.6497	1.2285
LOC_Os11g03520.1	Os11g0129500	GDSL-like lipase/acylhydrolase, putative, expressed	−0.7088	−0.2609	−0.4341	1.404
LOC_Os11g32650.1	Os11g0530600	chalcone synthase, putative, expressed	−0.5194	−0.5342	−0.7627	1.8165
Down-regulated in MB						
LOC_Os02g38130.1	Os02g0594800	no apical meristem protein, putative, expressed	0.3738	0.5906	0.2201	−1.1846
LOC_Os03g12510.1	Os03g0226200	non-symbiotic hemoglobin 2, putative, expressed	0.5177	1.17	0.1738	−1.8615
LOC_Os04g05650.1	Os04g0142400	expressed protein	0.3188	0.4501	0.5841	−1.353
LOC_Os07g11110.1	Os07g0212200	NAD dependent epimerase/dehydratase family protein	0.2827	1.0536	0.0270	−1.363
LOC_Os09g13940.1	Os09g0309700	AP2 domain containing protein, expressed	0.1881	0.8742	0.0502	−1.1123
LOC_Os10g22080.1	Os10g0365300	expressed protein	0.3461	1.1042	0.4549	−1.905
LOC_Os10g38340.1	Os10g0527400	glutathione S-transferase GSTU6, putative, expressed	−0.2856	1.7145	0.2405	−1.6695
LOC_Os11g03660.1	Os11g0131100	VQ domain containing protein, putative, expressed	0.3910	0.6612	0.3753	−1.4275
LOC_Os11g07960.1	Os11g0182200	transferase family protein, putative, expressed	0.1436	0.2447	0.7153	−1.1033
LOC_Os11g47560.1	Os11g0701500	glycosyl hydrolase, putative, expressed	0.2834	0.9313	0.0480	−1.2625

We also compared the expression levels of the putative mitochondrial genes between sporophytic and gametophytic CMS lines in rice. Fifty DEGs were found in both CMS types of rice (Additional file 8: Table S4). Alternative oxidase is a gene that is well known for its mitochondrial stress and it was over-expressed in the anthers of some CMS lines such as CW-CMS [22, 23]. In the present study, *AOX1a* (alternative oxidase 1a, LOC_Os04g51150) was observed to be up-regulated in all the CMS lines (Additional file 8: Table S4). It was interesting that 6 of the 50 DEGs, which encoded heat shock protein (HSP) and molecular chaperones were altered in their expression levels. Other genes showed the same pattern in both sporophytic and gametophytic CMS lines such as LEML3 (anther-specific LEM1 family protein, LOC_Os04g32700), NADP-dependent oxidoreductase (LOC_Os12g12580), proline oxidase (LOC_Os10g40360), and so on.

### Identification of WGCNA modules associated with male sterility related genes

**Fig. 8:**
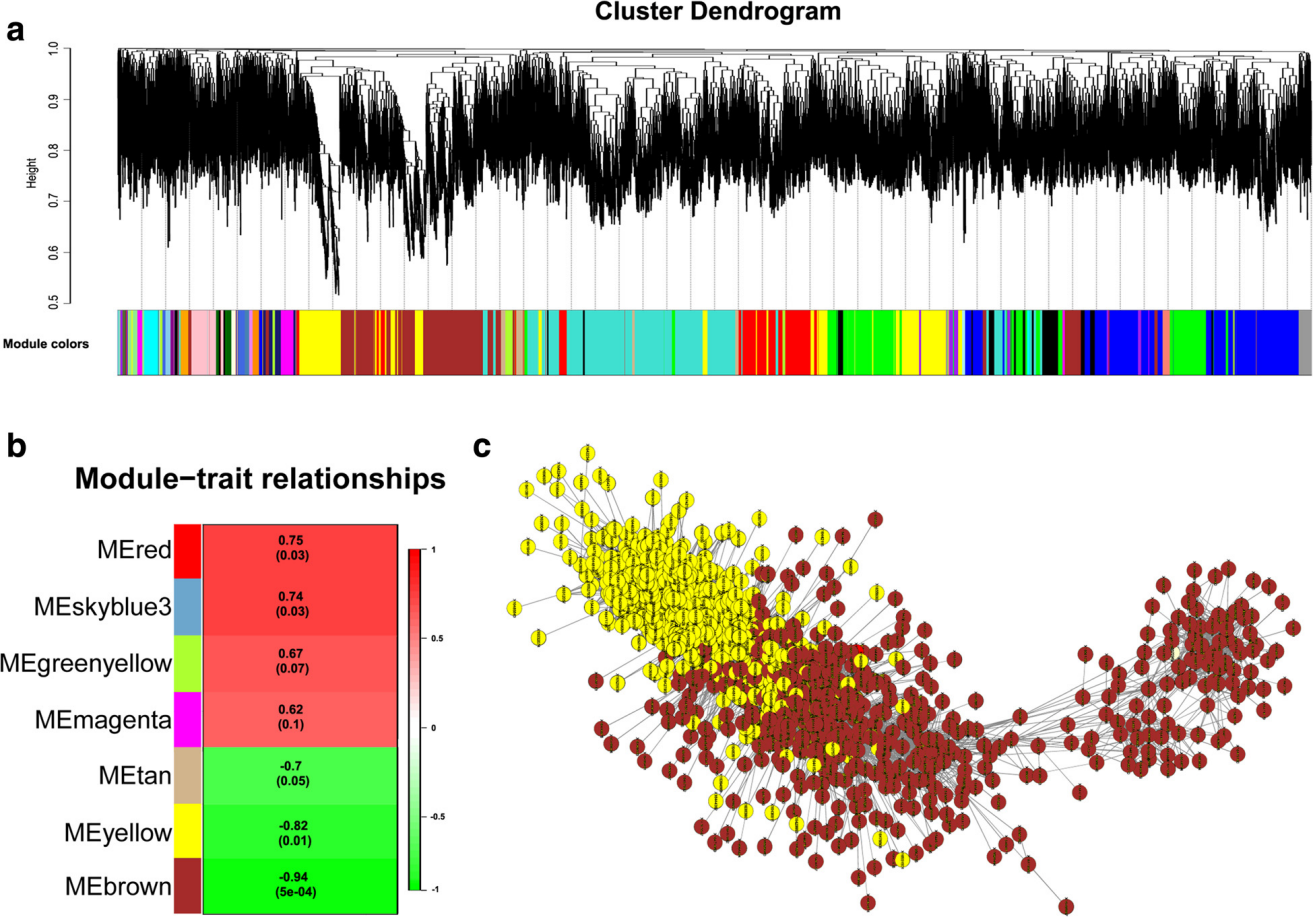
Weighted gene co-expression network analysis (WGCNA) of male sterility associated genes. **a** Hierarchical cluster tree showing the modules of co-expressed genes. The lower panel shows modules in designated colours. **b** Module-male sterility correlations and corresponding *p*-values (in parenthesis). The left panel shows seven module eigengenes (ME, red, skyblue3, greenyellow, magenta, tan, yellow, and brown). The right panel is a colour scale for module trait correlation from −1 to 1. **c** Cytoscape representation of co-expressed genes in ‘yellow’ and ‘brown’ modules

To access the genes associated with male sterility in the three CMS lines, a weighted gene co- expression network analysis (WGCNA) was performed with all the probes detected in the study, leading to 38 WGCNA modules (Fig. 8a). Analysis of the module-trait relationships revealed that the module ‘Brown’ (*r* = −0.94, *p* = 5e-04) and ‘yellow’ (*r* = −0.82, *p* = 0.01) were highly correlated with male sterility in the four samples (Fig. 8b and c). Cytoscape representation of these hub genes of two modules also indicated that they were highly associated with male sterility (Fig. 8c). Most of the DEGs mentioned above were included in the two modules (Additional file 9: Table S5). These hub genes included invertase/pectin methylesterase inhibitor (LOC_Os06g49760), WAX2 (LOC_Os10g33250), plasma membrane ATPase (LOC_Os12g44150), and receptor-like protein kinase 2 (LOC_Os01g07560) (Fig. 8c). Some of these genes, or their homologue genes, have been reported to be involved in pollen development, including male sterility protein (LOC_Os03g07140), aldehyde dehydrogenase (LOC_Os08g34210), ABC-2 type transporter (LOC_Os06g40550), and ribosome inactivating protein (LOC_Os07g37090) (Fig. 8c).

## Discussion

In this study, we developed a set of rice alloplasmic CMS lines by successive backcrossing, characterized their pollen phenotypes and analyzed the nuclear gene expression patterns. Comparison of the gene expression profiles of the alloplasmic CMS lines could be effective for elucidating the common and distinct features of each CMS type.

### Specific genes expressed in the alloplasmic CMS lines

There were unique features in the gene expression patterns of the three CMS lines. Cluster 1 was comprised of genes that were drastically down-regulated in XQZ-A/MB (Fig. 4), including glycosyl hydrolase (LOC_Os11g47570) and glycosyltransferase (LOC_Os07g10840). In Arabidopsis, glycosyl hydrolase family 17 genes were thought to be directly linked to pollen mother cell wall persistence and callose wall degradation. In this study, the down-regulated glycosyl hydrolase in XQZ-A/MB indicated that a pollen wall development defect might be associated with the CMS in the rice DA-CMS system.

Unlike the two CMS lines XQZ-A/MB and D62-A/MB, there were many genes that were over-expressed in ZS97-A/MB (Fig. 4). It is interesting that three genes associated with oxidative phosphorylation were up-regulated in ZS97-A/MB, including ATPase (LOC_Os05g02940 and LOC_Os07g09420) and the cytochrome c oxidase subunit (LOC_Os07g42910). Previous studies have reported that ATPase or cytochrome c oxidase dysfunction might be related to male sterility in many higher plants [24, 25]. It is possible that energy production dysfunction may lead to pollen abortion in these CMS lines. However, these genes that were over-expressed in ZS97-A/MB indicated that pollen abortion of WA-CMS was not due to an energy defect and exhibited a different mechanism from the HL-CMS system [26]. This hypothesis was demonstrated by the discovery of a new mitochondrial gene, *WA352*, which was found to inhibit *cox11* function and trigger premature tapetal programmed cell death, leading to pollen abortion in the rice WA-CMS line [9]. In the present study, another gene was characterized in cluster 14, metal transporter Nramp6 (LOC_Os07g15460), which was highly overexpressed in ZS97-A/MB (Figs. 4 and 7). It is well known that heavy metal treatment can induce ROS, and the highly expressed Nramp6 in ZS97-A/MB might be associated with ROS signalling in mitochondria.

On the other hand, unique features in the gene expression patterns of the D62-A/MB CMS line were also found. Some genes in cluster 2 were only up-regulated in D62-A/MB, including isocitrate lyase (LOC_Os07g34520), PDR ABC transporter (LOC_Os08g43120) and the thaumatin family protein (LOC_Os12g43450). Isocitrate lyase was reported to be active at specific stages of pollen development in *Brassica napus* [27], while thaumatin proteins are an allergen family in pollen [28]. Other genes in clusters 6 and 9 were down-regulated in D62-A/MB, such as OsIAA9 (LOC_Os02g56120), WRKY25 (LOC_Os08g13840), 3-ketoacyl-CoA synthase (LOC_Os03g12030), and so on. It has been reported that OsIAA9 can form heterodimers with OsIAA4 and OsIAA1, which play important roles in the cross-talk of auxin and brassinosteroid signalling pathways and plant morphogenesis [29]. The WRKY34 transcription factor has been demonstrated to be involved in pollen development and is regulated by the pollen-specific MIKC* class of MADS-domain transcription factors under cold stress [30]. In Arabidopsis, *KCS1*, encoding a 3-ketoacyl-CoA synthase, was demonstrated to affect the wax biosynthesis [31]. The DEGs indicated that some different processes participated in the CMS line D62-A/MB.

Interestingly, the CMS lines XQZ-A/MB and D62-A/MB shared many down-regulated genes. For instance, cluster 19 and cluster 21 included pollen ankyrin (LOC_Os06g03800), helix-loop-helix DNA binding protein (LOC_Os04g44600) and flavonol synthase/flavanone 3-hydroxylase (LOC_Os04g57160). Some lipid metabolism genes including fatty acid hydroxylase (LOC_Os03g03370) and wax synthase (LOC_Os05g48260) were also down-regulated in both XQZ-A/MB and D62-A/MB. It has been reported that flavanone 3-hydroxylase is necessary for the production of both flavonols and anthocyanins, while flavonols are required for functional pollen in maize [32]. As an integral membrane protein, wax synthase can catalyze fatty acids to wax-esters [33]. In Arabidopsis, fatty acid ω-hydroxylase was reported to be involved in suberin monomer biosynthesis and then affected cell wall formation. However, these genes were not significantly suppressed in the CMS line ZS97-A/MB, indicating that DA-CMS and D-CMS might have different regulation mechanisms for male sterility from that of WA-CMS in our study. Because there are few studies on DA-CMS and D-CMS, the specific nuclear expression patterns in this study suggested that pollen abortion of the two CMS lines might relate to the formation of pollen exine during pollen development.

### Candidate genes were essential for pollen development in rice

CMS is not only an ideal model to study cytoplasm-nuclear interactions but it can also discover genes that essential for pollen development by comprising gene expression between the CMS lines and its maintainer line. In this study, the DEGs in the three CMS lines were compared to previous expression profiles in five independent CMS lines [2]. A total of 303 DEGs were differentially expressed in all seven CMS lines, and 56 of them were down-regulated in CMS lines (Table 2 and Additional file 7: Table S3). These genes included male sterility protein (LOC_Os03g07140), ABC-2 type transporter (LOC_Os06g40550), and ribosome inactivating protein (LOC_Os07g37090). In Arabidopsis, male sterility protein 2 (MS2) has been predicted to encode a fatty acid reductase which can convert palmitoyl-acyl carrier protein to C16:0 alcohol and is required for pollen wall development [34–36]. In rice, the ABC-2 type transporter (LOC_Os06g40550), namely ABCG15, which encoded an ABC transporter protein, is essential for postmeiotic anther and pollen development and is proposed to play a role in the transport of rice anther cuticle and sporopollenin precursors [37–39]. The tapetum-specific gene *RA39* which has been reported to encode a ribosome-inactivating protein, played an important role in the regulation of tapetal development [40]. Therefore, the dramatically suppressed genes in many CMS lines might be candidate genes for anther and pollen development.

**Table 2:** Down-regulated genes in both three sporophytic and four gametophytic CMS lines compared with their maintainer lines

RGAP_ID	Annotation	XQZ-A/MB	ZS97-A/MB	D62-A/MB	CW_UN	W11_UN	LD_UN	BT_UN^a^
LOC_Os05g34700	GDSL-like lipase/acylhydrolase	−1.788	−1.863	−2.880	−1.248	−0.032	−2.767	−1.916
LOC_Os08g34210	aldehyde dehydrogenase	−1.465	−1.634	−2.162	−1.967	−0.009	−2.933	−2.633
LOC_Os12g12170	cytochrome b5-like Heme/Steroid	−1.569	−1.795	−2.366	−2.666	0.128	−3.665	−3.488
LOC_Os01g07560	receptor-like protein kinase 2 precursor	−2.441	−2.921	−3.254	−1.628	0.020	−6.419	−5.625
LOC_Os02g48730	rho GDP-dissociation inhibitor 1	−1.161	−1.433	−1.687	−0.912	0.215	−1.631	−1.846
LOC_Os03g14010	glycosyl hydrolase family 10 protein	−1.748	−2.064	−2.538	−2.496	−0.866	−1.624	−2.753
LOC_Os06g48170	glycosyl hydrolases family 16	−1.098	−1.348	−1.576	−1.896	−0.906	−2.381	−1.907
LOC_Os06g49760	invertase/pectin methylesterase inhibitor	−1.232	−1.755	−2.007	−1.332	0.187	−1.885	−1.748
LOC_Os12g13930	3-oxoacyl-reductase	−4.391	−3.624	−4.626	−1.837	−0.111	−6.379	−5.131
LOC_Os09g36860	acyl carrier protein	−1.250	−1.209	−1.485	−1.811	−0.567	−2.923	−2.586
LOC_Os06g40550	ABC-2 type transporter	−3.262	−3.257	−3.347	−2.983	−0.543	−9.950	−7.678
LOC_Os04g45960	OsSub42 - Putative Subtilisin	−5.103	−4.710	−5.378	−2.310	−0.430	−8.942	−9.165
LOC_Os06g12330	amino acid transporter	−2.746	−2.574	−3.035	−4.736	−0.043	−5.436	−5.730
LOC_Os04g48210	cytochrome P450	−4.450	−4.365	−4.178	−2.146	−0.027	−11.565	−10.364
LOC_Os03g08790	aspartic proteinase nepenthesin	−1.534	−1.483	−1.476	−2.025	−0.313	−9.300	−9.180
LOC_Os03g07140	male sterility protein	−3.090	−2.931	−4.804	−1.524	−0.169	−2.822	−2.552
LOC_Os07g37090	ribosome inactivating protein	−3.422	−4.056	−4.848	−3.277	0.688	−10.619	−9.790
LOC_Os02g02820	TDR	−1.918	−2.015	−2.442	−0.676	−0.459	−0.759	−0.686

^a^The microarray data of GSE18057 (Fujii et al., [2])

On the basis of Pearson’s correlation coefficient for the genes, a co-expression network of all the probesets in all three CMS lines demonstrated that some hub genes play important roles in pollen development, including aldehyde dehydrogenase (LOC_Os08g34210), receptor-like protein kinase 2 (LOC_Os01g07560), ABC-2 type transporter (LOC_Os06g40550), and ribosome inactivating protein (LOC_Os07g37090) (Fig. 8 and Additional file 9: Table S5). It has been reported that aldehyde dehydrogenase (ALDH) activity is important for pollen tube growth in tobacco and is required to restore male fertility in T cytoplasm maize [41, 42]. Further functional studies on these genes will elucidate how they are involved in pollen development.

During pollen development, one fundamentally important event is the deposition of the pollen wall, which is necessary for pollen protection, dispersal, and pollen-stigma recognition [43, 44]. At the tetrad stage, a microspore-derived cellulosic primexine is synthesized by the developing haploid microspores. A thick exine, whose components are synthesized by sporophytes, is deposited on the outer surface of the primexine largely after release of free microspores [45]. In later pollen developmental stages, the tapetum produces and secretes lipidic components of pollen coat/tryphine into exine cavities [43, 45]. Since many down-regulated DEGs in the sporophytic CMS lines were found to be involved in tapetum or wall formation in this study, their suppressed expression might be related to male sterility and these genes might be essential for pollen development.

## Conclusions

In this study, the transcript profiles of three alloplasmic sporophytic CMS lines were compared with their maintainer line Meixiang B using microarray. A total of 622 differentially expression genes (DEGs) in each of the three CMS lines were identified and 114 DEGs were shared in the three CMS lines. GO and Mapman analysis indicated that the shared DEGs were mainly involved in lipid metabolic and cell wall organization. Compared with the gene expression of sporophytic and gametophytic CMS lines, 303 DEGs were identified and 56 of them were down-regulated in all the CMS lines. These down-regulated DEGs were shown to be involved in tapetum or cell wall formation and their suppressed expression might be related to male sterility. Weighted gene co-expression network (WGCNA) analysis revealed two modules that were significantly associated with male sterility and many hub genes that were differentially expressed in the CMS lines. These provide a rich resource for further functional research on pollen development in rice.

## Methods

### Plant materials

The DA-CMS, WA-CMS, and D-CMS lines were backcrossed with the maintainer line Meixiang B (MB) seven times (Additional file 10: Figure S5). The three CMS lines XQZ-A/MB (DA-CMS), ZS97-A/MB (WA-CMS) and D62-A/MB (D-CMS) were conserved in our lab and were backcrossed in the field of Lingshui, Hainan, China (18°48' N; 110°02' E). Finally, these rice alloplasmic CMS lines were grown in a rice paddy in the fields of Wuhan University (30°34' N; 114°17' E) under natural conditions. The rate of nuclear substitution by the MB genome was determined using 54 simple sequence repeat (SSR) markers (Additional file 11: Table S6). And the rate was 97.6 % for XQZ-A/MB, 86.0 % for ZS97-A/MB and 88.2 % for D62-A/MB. Therefore, these three lines were near isogenic lines with the nuclear genome of MB.

### DNA extraction and Southern blotting analysis

Total DNA was isolated from XQZ-A/MB, D62-A/MB, ZS97-A/MB and MB calluses by the CTAB method. Approximately 20 μg of DNA was restricted by *Eco*R I, *Hin*d III, *Bam*H I, and *Xba* I. The restricted fragments were separated by electrophoresis using 0.8 % (w/v) agarose gels in 0.5 × TBE buffer. Then the DNA was transferred to Nytran N Nylon membranes (Schleicher and Schuell, Keene, USA) and cross linked to the membrane by use of UV crosslinker CL-1000 (UVP, Upland, USA). Probes were designed from 6 different mitochondrial genes (Additional file 11: Table S6). Digoxigenin-labeled DNA fragments were obtained by use of PCR DIG Labeling Mix (Roche Diagnostics, Basel, Switzerland). After hybridization of the probes to the membranes, probe residues were washed and anti-DIG AP Fab fragments (Roche Diagnostics, Basel, Switzerland) were used for detection.

### Microscopic observation of pollen morphology

The florets at different developmental stages during anther development were fixed in Carnoy's Fluid (ethanol: acetic acid = 3:1), and then stored at 4 °C until observation. The anther morphology was observed by stereomicroscope. The pollen grains were stained in 1 % (w/v) iodine-potassium iodide solution and improved carmine solution and then observed by ordinary optical microscope. A coupled CCD camera DP80 (Olympus, Japan) was used to take photos.

### Total RNA isolation and cDNA microarray hybridization

Anthers of the three CMS lines and MB were harvested at the uninucleate microspore stage, frozen in liquid nitrogen, and then kept at −80 °C. Total RNA was extracted using TRIzol reagent (Invitrogen) according to the manufacturer’s instructions. The cDNA microarray hybridization was performed with the GenAtlas Rice (Cn) Gene 1.1 ST Array Strip (ssp. *Indica*) (CapitalBio Corp.). An aliquot of 10 μg total RNA was used to produce biotin-labelled cDNA according to the manufacturer’s instructions. Biotin- labeled cDNA was then hybridized with the microarray at 42 °C for 2 h. After the washing and drying steps, the microarrays were scanned and analyzed by the GeneAtlas™ Imaging Station. All the stages/lines combinations had two biological replicates.

### Microarray data analysis

The data were normalized by Robust Multichip Analysis Robust Multichip Analysis (RMA) and then log transformed. Differentially expressed genes (DEGs) were identified using *t* test and multiple test corrections were performed using the False Discovery Rate (FDR) [46]. Genes with FDR <0.01 and a fold change greater than or equal to two were identified as DEGs. The expression value was defined as the average of the two independent hybridizations for each of the CMS lines, and statistically significant changes in expression were evaluated by one-way ANOVA (*p* < 0.01) as described [47]. After normalization, hierarchical clustering and k-means clustering of the expression patterns were performed by Multiexperimental Viewer v4.7 [48]. The expression values were converted to a log2 scale with previous reported transcriptome data for GSE18057 [2] in the NCBI to compare the expression patterns between sporophytic and gametophytic CMS lines.

### RT-PCR analysis and quantitative RT-PCR

The DEGs were selected to be verified by RT-qPCR, using the same RNA that was used for the microarray. The first strand cDNA was synthesized from 5 μg total RNA from each sample using RevertAid First Strand cDNA Synthesis Kit (Fermentas, USA) according to the manufacturer’s instructions. Rice *actin1* gene was used as the internal control for RT-qPCR analysis. All primers for the candidate genes and *actin1* were designed by the Primer3 program (http://redb.ncpgr.cn/modules/redbtools/primer3.php) and are shown in Additional file 11: Table S6. And then RT-qPCR was performed with an ABI StepOne Real-Time PCR System (Applied Biosystems, USA) using a SYBR Premix Ex Taq Kit (TaKaRa, Japan). The relative expression level was normalized and quantified using a ^△△^CT method. The PCRs were conducted with the following program: an initial denaturation at 95 °C for 30s, followed by 40 cycles of 95 °C for 10 s, 56 °C for 30 s, and 72 °C for 15 s. After the amplification steps, the melting curve was determined for each primer pair to verify that only one specific product had been amplified. Three replicates were performed for each sample. The significant differences of expression level between the CMS line and maintainer line were evaluated using Student's *t* test (* *p* < 0.05; ** *p* < 0.01).

### Inferring sterility associated co-expression gene network modules

The highly co-expressed gene modules were inferred from all the probe sets using the weighted gene co- expression network analysis (WGCNA) package in R [49, 50]. An adjacency matrix was generated based on a pairwise Pearson’s correlation coefficient (PCC) between two genes across all samples. WGCNA network construction and module detection was conducted using an unsigned type of topological overlap matrix (TOM), a power β of 14, a minimal module size of 30, and a branch merge cut height of 0.25. The module eigengene (ME, the first principal component of a given module) value was calculated and used to evaluate the association of modules with male sterility in the 4 samples. The most significant module was visualized using Cytoscape 3.1.1 [51] and was also analyzed using the Cytoscape plugin Network Analyzer [52].

## Abbreviations

ALDH, aldehyde dehydrogenase; ANOVA, analysis of variance; CHS, chalcone synthase; CMS, cytoplasmic male sterility; D, Dissi; DA, dwarf-wild-abortive; DEGs, differentially expression genes; FDR, false discovery rate; GO: gene ontology; HL, Honglian; HSP, heat shock protein; ID, Indonesia paddy; MB, Meixiang B; MS2, male sterility protein 2; ORF, open reading frame; PMEI, invertase/pectin methylesterase inhibitor; RAP-DB, rice annotation project-database; RFLP, restriction fragment length polymorphism; RGAP, rice genome annotation project; RMA, robust multichip analysis; ROS, reactive oxygen species; RT-qPCR, reverse transcriptase quantitative PCR; SSR, simple sequence repeat; WA, wild abortive; XQZ-A, Xieqingzao-A; ZD, Zidao; ZS97-A, Zhenshan97-A

## Additional files

Additional file 1: Figure S1. Cytological observation of pollen morphology. Upper, the anther phenotypes of the three CMS lines (A, XQZ-A/MB, B, ZS97-A/MB, C, D62-A/MB) and the maintainer line Meixiang B (D), bar =1 mm. Lower, 1 % I2-KI staining of the pollen grains of the three CMS lines (E, XQZ-A/MB, F, ZS97-A/MB, G, D62-A/MB) and the maintainer line Meixiang B (H), bar =20 μm. (PDF 147 kb) Additional file 2: Figure S2. Venn diagram of DEGs in the three CMS lines. Among these genes, 114 DEGs were shared in all the CMS lines. The numbers of specifically expressed genes in each CMS line were 45 (XQZ-A/MB), 158 (ZS97-A/MB) and 219 (D62-A/MB), respectively. (PDF 49 kb) Additional file 3: Figure S3. All the expression patterns of the 622 DEGs in the three CMS lines and the maintainer line MB. (PDF 68 kb) Additional file 4: Table S1. The expression patterns and annotations of 622 DEGs identified in each of the three CMS lines. (XLSX 141 kb) Additional file 5: Table S2. The shared 114 DEGs in the three CMS lines compared with maintainer line (MB). (XLSX 186 kb) Additional file 6: Figure S4. GO analysis of the 622 DEGs in the three CMS lines. (A) GO enrichment of biological process; (B) GO enrichment of Molecular function. (PDF 316 kb) Additional file 7: Table S3. The down-regulated and up-regulated DEGs in both sporophytic and gametophytic CMS lines. (XLS 152 kb) Additional file 8: Table S4. The shared mitochondrial DEGs in both sporophytic and gametophytic CMS lines. (XLS 254 kb) Additional file 9: Table S5. Genes in the two modules 'yellow' and 'brown' were analyzed by weighted gene co- expression network analysis (WGCNA). (XLSX 315 kb) Additional file 10: Figure S5. The process of construction of alloplasmic male sterile lines. (PDF 64 kb) Additional file 11: Table S6. List of primers used for SSR, RT-qPCR and Southern blotting analysis. (XLSX 101 kb)
