# Supplementary material for: Comparative transcript profiling of alloplasmic male-sterile lines revealed altered gene expression related to pollen development in rice (Oryza sativa L.)
Source: BMC Plant Biol. 2016 Aug 5;16:175. doi: 10.1186/s12870-016-0864-7 (PMC4974769; doi:10.1186/s12870-016-0864-7)
Supplement: Additional file 1: Figure S1. — Cytological observation of pollen morphology. Upper, the anther phenotypes of the three CMS lines (A, XQZ-A/MB, B, ZS97-A/MB, C, D62-A/MB) and the maintainer line Meixiang B (D), bar =1 mm. Lower, 1 % I2-KI staining of the pollen grains of the three CMS lines (E, XQZ-A/MB, F, ZS97-A/MB, G, D62-A/MB) and the maintainer line Meixiang B (H), bar =20 μm. (PDF 147 kb) [file 12870_2016_864_MOESM1_ESM.pdf]

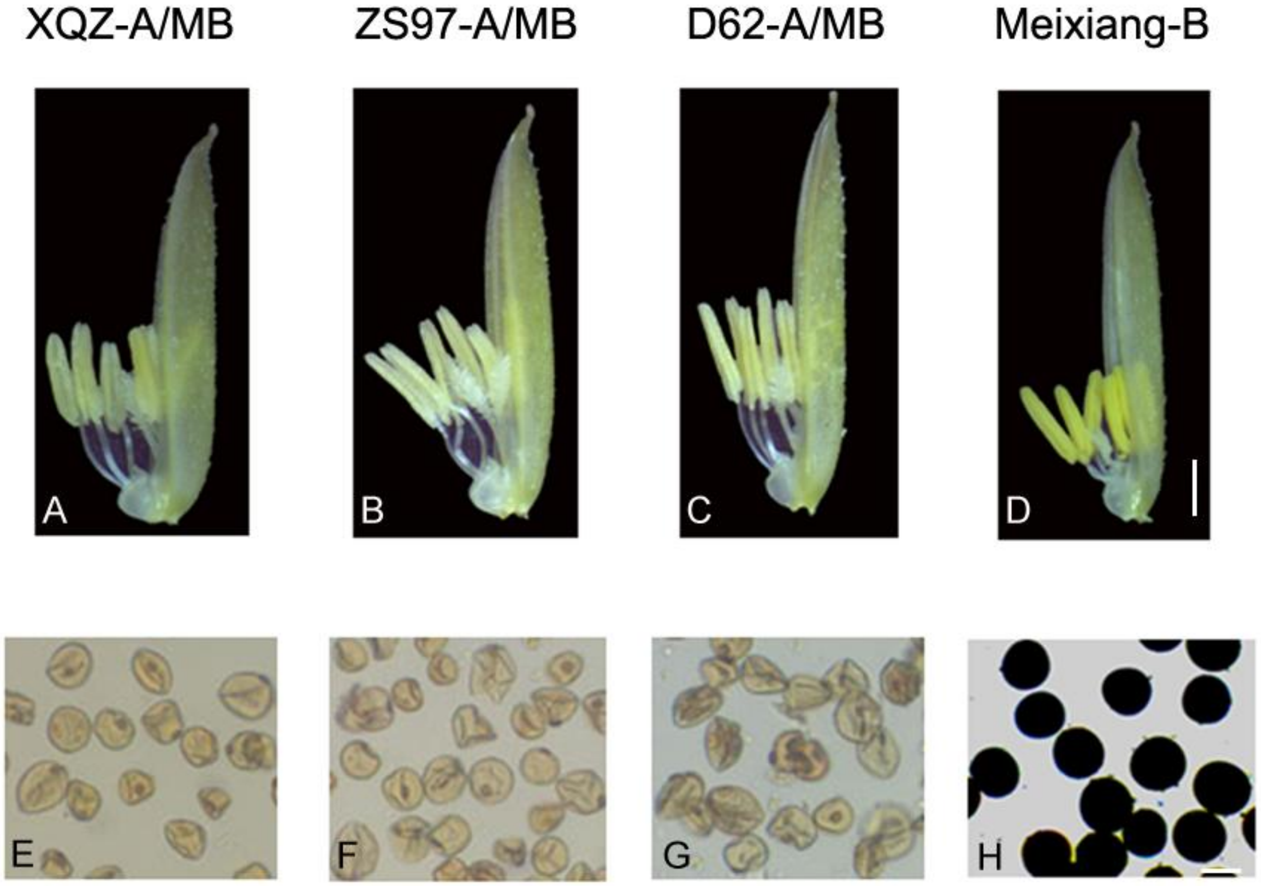

Fig. S1. **Cytological observation of pollen morphology.** Upper, the anther phenotypes of the three CMS lines (A, XQZ-A/MB, B, ZS97-A/MB, C, D62-A/MB) and the maintainer line Meixiang B (D), bar =1mm. Lower, 1% I_2-KI staining of the pollen grains of the three CMS lines (E, XQZ-A/MB, F, ZS97-A/MB, G, D62-A/MB) and the maintainer line Meixiang B (H), bar =20μm.
