# Supplementary material for: Comparative transcript profiling of alloplasmic male-sterile lines revealed altered gene expression related to pollen development in rice (Oryza sativa L.)
Source: BMC Plant Biol. 2016 Aug 5;16:175. doi: 10.1186/s12870-016-0864-7 (PMC4974769; doi:10.1186/s12870-016-0864-7)
Supplement: Additional file 2: Figure S2. — Venn diagram of DEGs in the three CMS lines. Among these genes, 114 DEGs were shared in all the CMS lines. The numbers of specifically expressed genes in each CMS line were 45 (XQZ-A/MB), 158 (ZS97-A/MB) and 219 (D62-A/MB), respectively. (PDF 49 kb) [file 12870_2016_864_MOESM2_ESM.pdf]

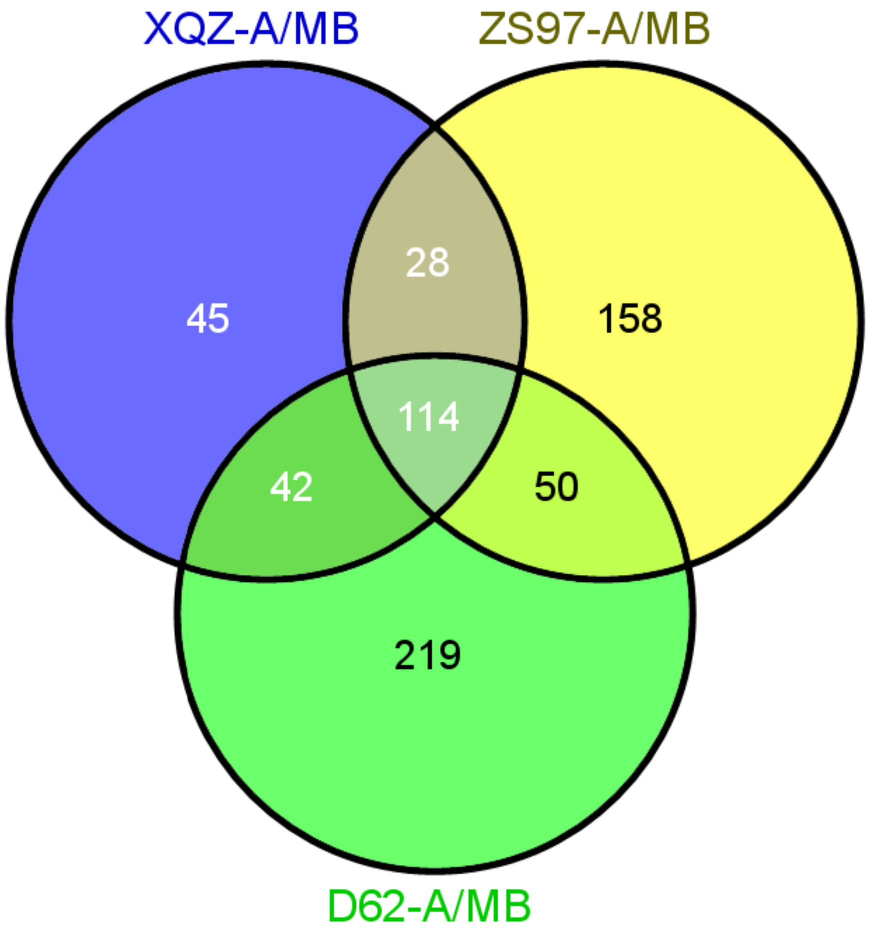

Fig. S2. Venn diagram of DEGs in the three CMS lines. Among these genes, 114 DEGs were shared in all the CMS lines. The numbers of specifically expressed genes in each CMS line were 45 (XQZ-A/MB), 158 (ZS97-A/MB) and 219 (D62-A/MB), respectively.
