# Supplementary figures and images for: Comparative transcript profiling of alloplasmic male-sterile lines revealed altered gene expression related to pollen development in rice (Oryza sativa L.)
Source: BMC Plant Biol. 2016 Aug 5;16:175. doi: 10.1186/s12870-016-0864-7 (PMC4974769; doi:10.1186/s12870-016-0864-7)

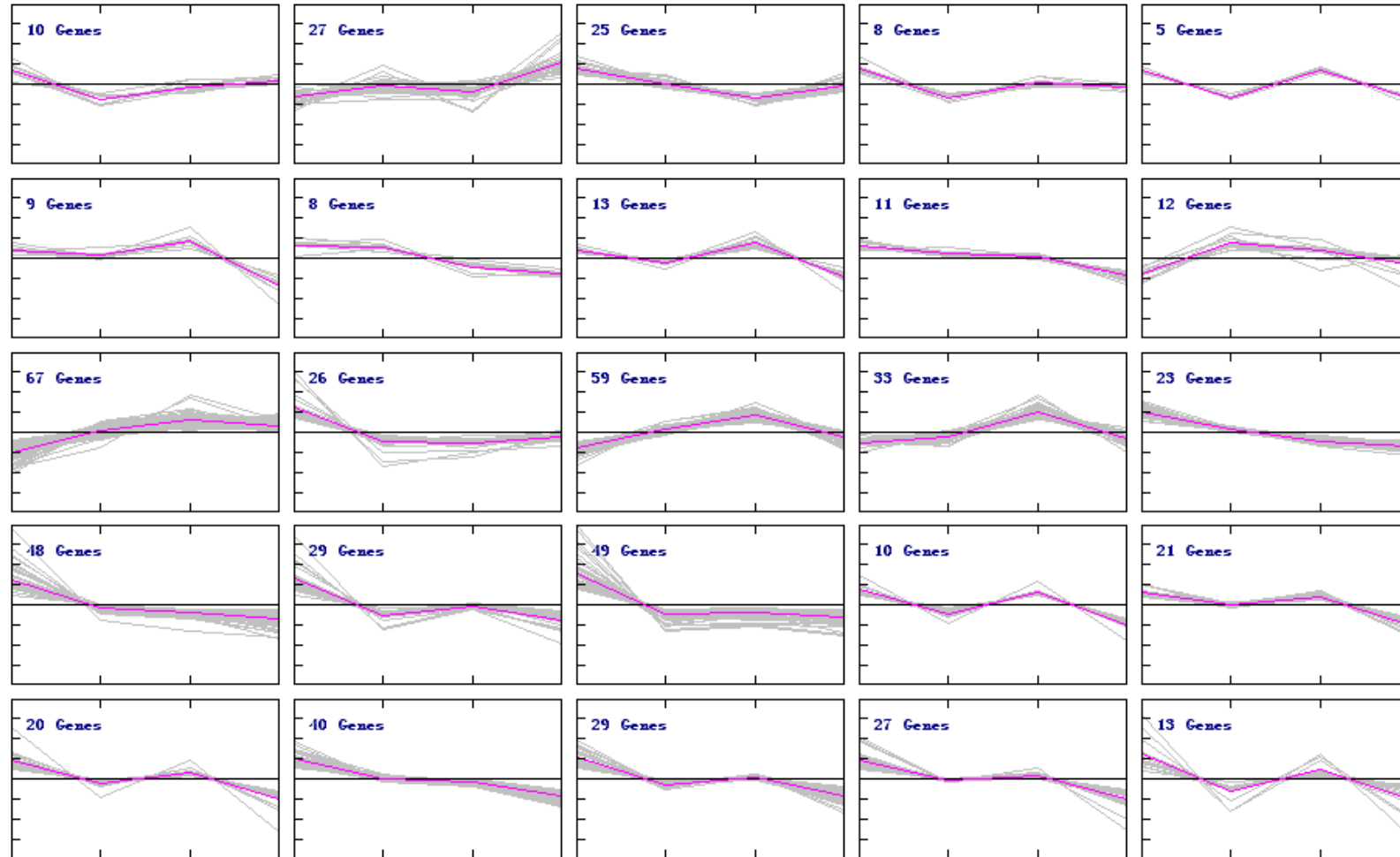

**Fig.S3.** All the expression patterns of the 622 DEGs in the three CMS lines and the maintainer line MB.

Supplement: Additional file 3: Figure S3. — All the expression patterns of the 622 DEGs in the three CMS lines and the maintainer line MB. (PDF 68 kb) [file 12870_2016_864_MOESM3_ESM.pdf]

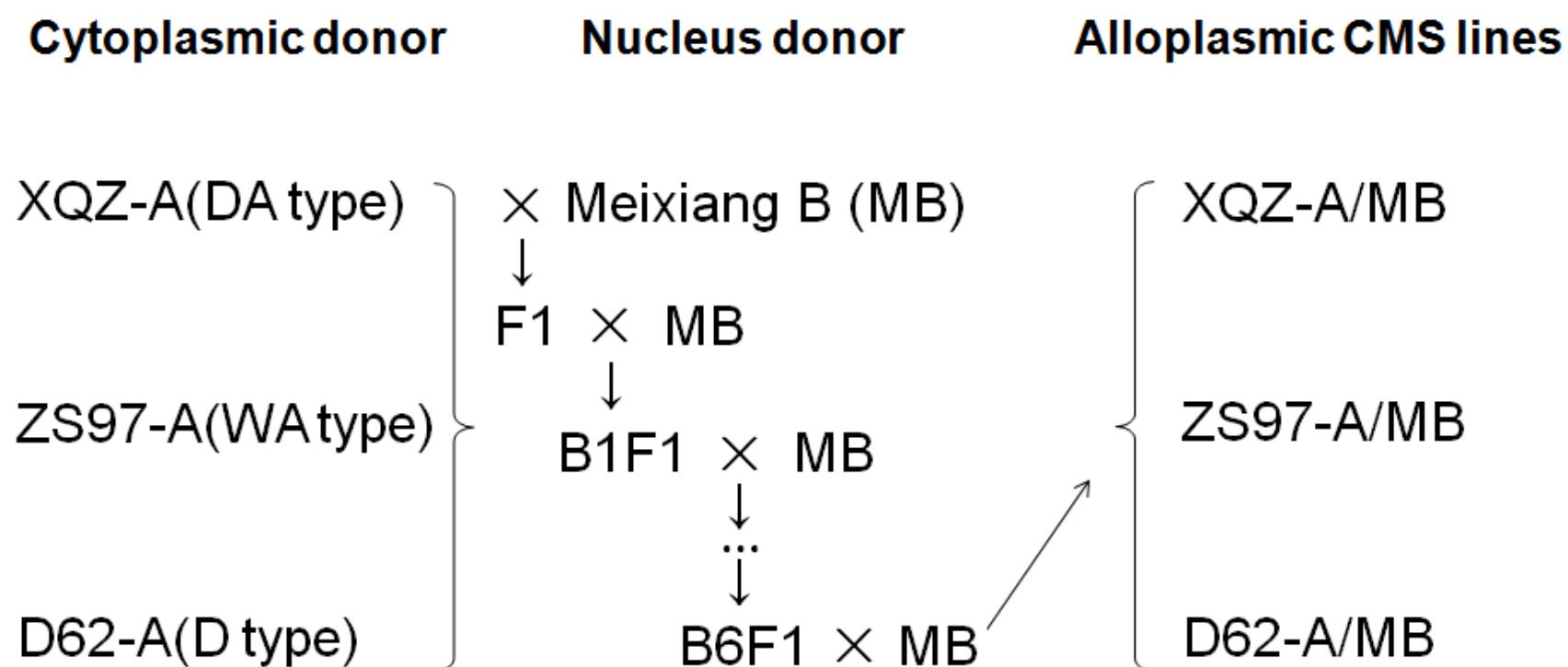

**Fig. S5.** The process of construction of alloplasmic male sterile lines.

Supplement: Additional file 10: Figure S5. — The process of construction of alloplasmic male sterile lines. (PDF 64 kb) [file 12870_2016_864_MOESM10_ESM.pdf]
