# Supplementary material for: Comparative transcript profiling of alloplasmic male-sterile lines revealed altered gene expression related to pollen development in rice (Oryza sativa L.)
Source: BMC Plant Biol. 2016 Aug 5;16:175. doi: 10.1186/s12870-016-0864-7 (PMC4974769; doi:10.1186/s12870-016-0864-7)
Supplement: Additional file 6: Figure S4. — GO analysis of the 622 DEGs in the three CMS lines. (A) GO enrichment of biological process; (B) GO enrichment of Molecular function. (PDF 316 kb) [file 12870_2016_864_MOESM6_ESM.pdf]

A

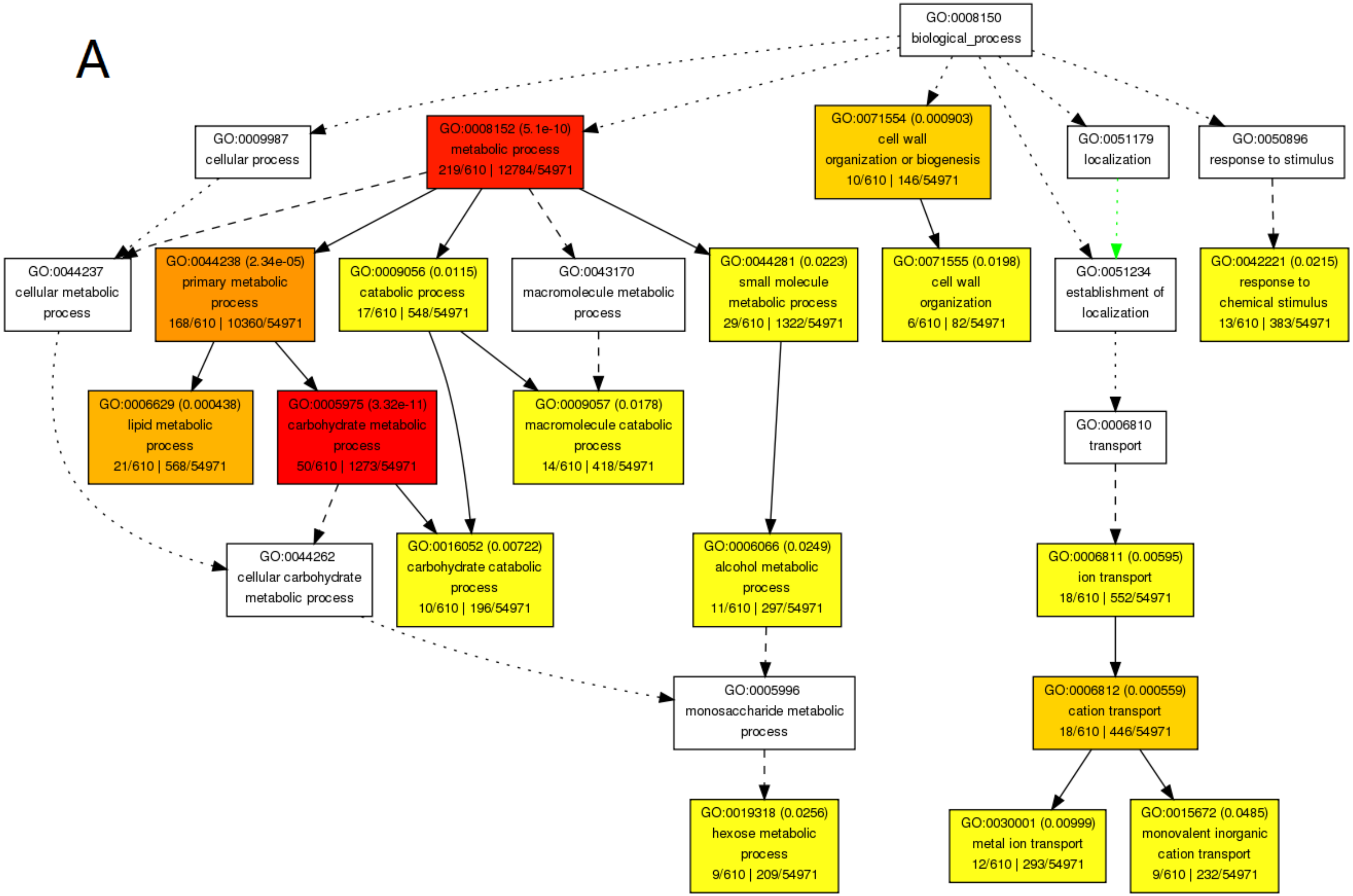

GO:0008150
biological_process
GO:0009987
cellular process
GO:0008152 (5.1e-10)
metabolic process
219/610 | 12784/54971
GO:0071554 (0.000903)
cell wall
organization or biogenesis
10/610 | 146/54971
GO:0051179
localization
GO:0050896
response to stimulus
GO:0044237
cellular metabolic
process
GO:0044238 (2.34e-05)
primary metabolic
process
168/610 | 10360/54971
GO:0009056 (0.0115)
catabolic process
17/610 | 548/54971
GO:0043170
macromolecule metabolic
process
GO:0044281 (0.0223)
small molecule
metabolic process
29/610 | 1322/54971
GO:0071555 (0.0198)
cell wall
organization
6/610 | 82/54971
GO:0051234
establishment of
localization
GO:0042221 (0.0215)
response to
chemical stimulus
13/610 | 383/54971
GO:0006629 (0.000438)
lipid metabolic
process
21/610 | 568/54971
GO:0005975 (3.32e-11)
carbohydrate metabolic
process
50/610 | 1273/54971
GO:0009057 (0.0178)
macromolecule catabolic
process
14/610 | 418/54971
GO:0006810
transport
GO:0044262
cellular carbohydrate
metabolic process
GO:0016052 (0.00722)
carbohydrate catabolic
process
10/610 | 196/54971
GO:0006066 (0.0249)
alcohol metabolic
process
11/610 | 297/54971
GO:0006811 (0.00595)
ion transport
18/610 | 552/54971
GO:0005996
monosaccharide metabolic
process
GO:0006812 (0.000559)
cation transport
18/610 | 446/54971
GO:0019318 (0.0256)
hexose metabolic
process
9/610 | 209/54971
GO:0030001 (0.00999)
metal ion transport
12/610 | 293/54971
GO:0015672 (0.0485)
monovalent inorganic
cation transport
9/610 | 232/54971

Fig. S4.

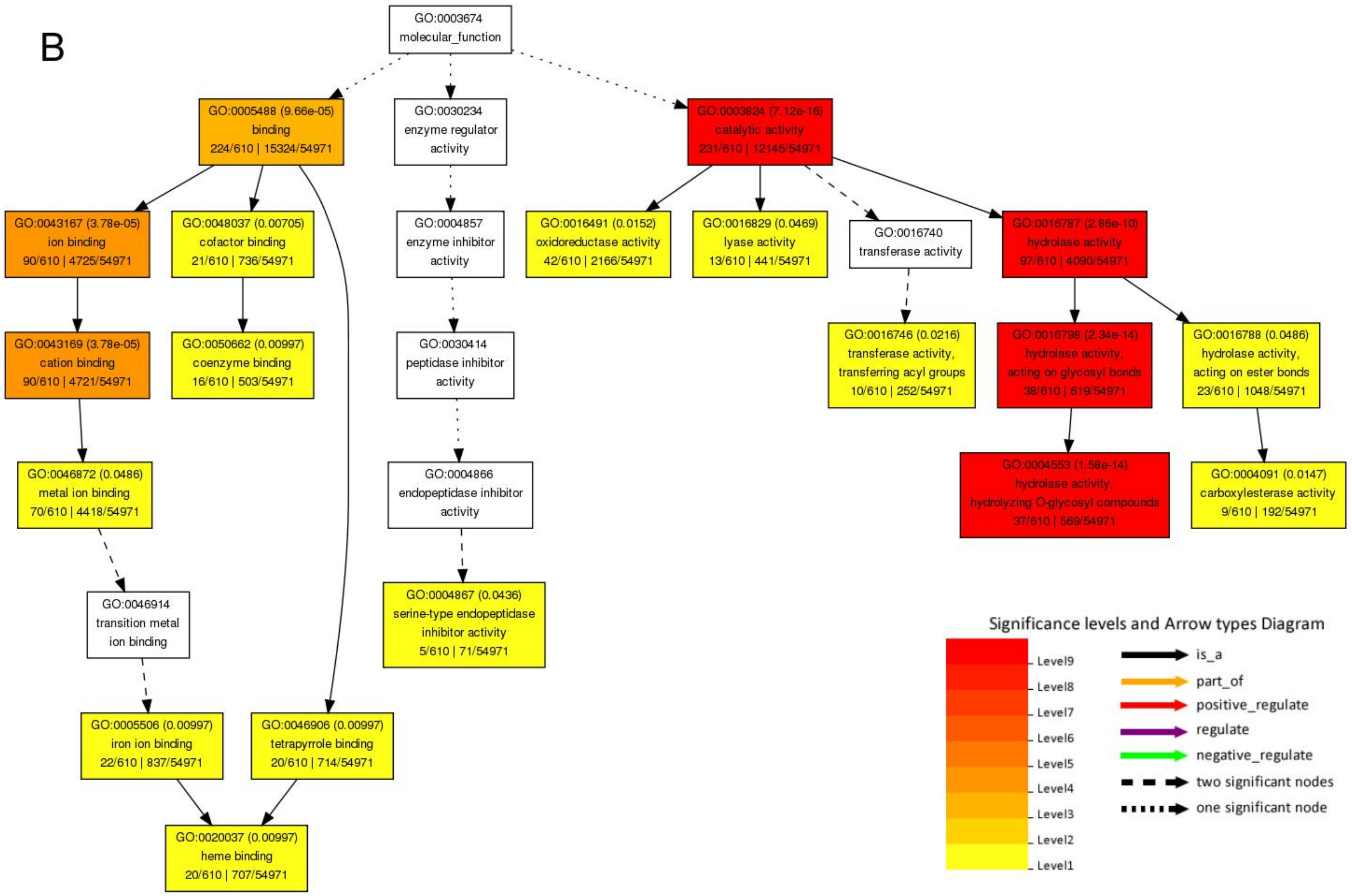

Fig. S4. GO analysis of the 622 DEGs in the three CMS lines. (A) GO enrichment of biological process; (B) GO enrichment of Molecular function.
